# Address to the Graduating Class, University of Buffalo1Delivered Commencement Day, May 27, 1910.

**Published:** 1910-07

**Authors:** Matthew D. Mann

**Affiliations:** Buffalo, N. Y.; Dean and Professor of Obstetrics and Gynecology in the Medical Department of the University


／# BUFFALO MEDICAL JOURNAL.

VOL. LXV. JULY, 1910. No. 12

### ORIGINAL COMMUNICATIONS.

### Address to the Graduating Class, University of Buffalo[1]

By MATTHEW D. MANN, A.M., M.D., Buffalo. N. Y.

Dean and Professor of Obstetrics and Gynecology in the Medical Department of the University.

*Ladies and Gentlemen of the Graduating Class:*

I CONGRATULATE you most heartily on the freedom which comes to you today. At last you have escaped from the bondage of teachers and professors,—you are about to begin your real life's work. Henceforth you will be your own masters, obeying no one's beck and call. Most of your life so far has been under tutelage. During childhood you met it in the home and in the school, perhaps after that you worked for a while in some subordinate business position; then came the professional school with its hard, grinding work,—I know of none harded,—very little time for play or amusement, with the terrors of frequent examinations hanging over you. Now that is all passed,—one more examination, and that not a severe one (not so severe as it ought to be, and certainly possessing no terrors for the graduates of the University of Buffalo), and you will be free.

While I congratulate you, I also sympathise with you, for, from this time on, you will be compelled to stand alone. Henceforth there will be no guiding hand to lead. The battles of life are real and earnest. Some are victors; some fail. The terrors of failure may hang over you, but not if you are of the right stuff. Success can be attained if you have chosen aright, and if you work in the right spirit.

Perhaps you would like me to tell you how to ensure success. I wish I could do it. The power to succeed is one of those intangible, imponderable forces which cannot be recognised, let alone described. Circumstances over which we have no control often have a deciding influence. Then, again, the ability to see and embrace an opportunity is very important. Remember what the poet says:

> "There is a tide in the affairs of men,
> Which, taken at the flood, leads on to fortune.
> Omitted, all the voyage of their life,
> Is bound in shallows and in miseries."

1. Delivered Commencement Day, May 27, 1910.

Still, there are two factors which go far toward the achievement of success. They are earnestness and honesty. Need I describe them? You know what I mean. No man nor woman can win except by doing what they have to do, with their might. No man nor woman can achieve a lasting satisfying success,—a success which leaves nothing to regret, leaves no sting nor remorse,—unless they get it by honest methods. Don't envy his apparent good fortune to the charlatan, the grafter, the schemer, the briber or the bribed, for it is sure, sooner or later, to turn to apples of Sodom on his lips.

Now, with this fatherly advice, I am going to change the subject from yourselves to myself. I do not mean that I am going to talk about myself very much, but, rather, about my experiences in a rather long and certainly very interesting life.

With the conclusion of this address, my duties as professor of obstetrics and gynecology in the Medical Department of the University of Buffalo, come to an end. It has been one of the most important relations of my life, and one of the most enjoyable. It is what brought me to Buffalo, and so cast my lines in pleasant places. Whether I have properly fulfilled my duties, I must leave to my colleagues, my associates and my students to decide. I have done the best I could. To my colleagues in the permanent faculty, I wish to express my sincerest thanks for all their courtesies and forbearances. I have found in their number some of the truest friends and some of the noblest men with whom it has been my privilege to be brought into close association. I shall always consider it an honor to have been in the same faculty with the late Drs. Moore, Thomas F. Rochester and Miner. They were men whose like is rarely seen, and their influence on my life, especially the influence of Dr. Rochester, with whom I was associated for a longer period, and whom I succeeded as dean, has been greatly to my advantage. They were noble men, who always stood for what was right, and whose example to all the younger men of the day was most illustrious. Long be the day before their memory fades!

Of the faculty of today, with whom I have been so long associated, I do not dare to speak, because I could not do it without emotion. They are my friends. What more can I say? In the 28 years in which I have been in the faculty,—during 23 years of which I have presided over its deliberations,—there has never been a serious disagreement or anything approaching a quarrel. All have been willing to sink their individual interests and opinions for the good of the institution, having only that in view. For years they have worked faithfully and long, with scant reward, sometimes even giving money or making other sacrifices, to make improvements from which they could never hope to reap any personal advantage. Nobody who has not been on the inside can ever appreciate the difficulties under which we have worked, or know how much we have accomplished with scant resources. The faculty has always been in the lead in trying to extend the curriculum and to raise the standard, both for entrance and for graduation, and especially for the license to practise.

The first steps for the creation of a state examining board were made by the members of this faculty, and deputations have been sent to Albany to try and raise the standard of both the entrance and the licensing examinations.

To the associate faculty, we, the members of the permanent faculty, owe a deep debt of gratitude. Working as they have, without pay, and often without hope of reward other than the good that might come to them by study and intellectual effort in teaching, they deserve all praise. I take great pleasure in behalf of the permanent faculty and for myself, in bestowing it.

It has been easy to work together when all were embued with a spirit of self-sacrifice and a desire for advancement. Notwithstanding this, we have often met with undeserved criticism, even with reproach. I think, however, that we have a reward in seeing the general respect in which our college is held and in watching the brilliant careers of some of our medical graduates.

It is from the faculty of the Medical Department, that the suggestions came for the building up of a Greater University. From the same source came, if not the initiative, certainly much of the effort which resulted in the founding of the Departments of Pharmacy and Dentistry. It is from the work of the same men that there resulted the union of the Medical Department of the University of Buffalo and the like Department of Niagara University.

When a traveler comes to the end of a long stretch of road, he is apt to turn around and look back over the country through which he has passed. I have been teaching medicine for 36 years, and have been connected with the faculties of three great universities. Much has happened in this time, and I may perhaps be pardoned if I take the time to tell you a little of what I have seen.

If I could, in a prenatal state, have selected that period in the world's history when I would have chosen to be born, I would have fixed it just when it happened. I do not believe in the whole history of mankind there has been a more interesting absorbing, yes, even exciting period in which to have lived, than that comprised in the last fifty years. I do not believe that ever again will there be another half century so full of, so crowded with, interest. In some respects it has been the most important in the history of the human race. We cannot tell what the future has in store, and you may see just as striking and startling changes as I have, but it hardly seems possible, for we, in one generation, have, in matters scientific, stepped from darkness into light. More has been accomplished in that time in the way of material advancement and in the attainment of useful knowledge, than in the whole period which has elapsed since the fall of Troy. Nearly the whole body of what we know as modern science has been achieved within that time. The foundations only had been laid by Bacon, Newton, and Franklin, and the discoverers of the telescope and the microscope. Chemistry, especially organic chemistry, is not fifty years old. The science of electricity is younger still, while the whole subject of physics has been put on a new basis and immeasurably increased within very recent times.

It is but natural, then, that science having made such marvelous advances, its application to the solution of every day problems should follow. Thus, we have the wonderful improvements in manufacture and agriculture, in thought transmission, in transportation,—the harnessing of the natural forces of nature to do our bidding.

From these advances have come wonderful sociological developments,—the founding of republics, as a result of a more general diffusion of knowledge; the building of great cities, which before would have been impossible; the taming of continents previously given up to savagery or nature. I remember when the United States was made up largely of the Northwest Territory and the Great American desert; when South America was a continent of warring republics and one monarchy, stretched along the coast of a great continent; when all of Central Africa was an unknown land, and Siberia was supposed to be an uninhabitable wilderness; when Australia was a penal colony; when China and Japan were almost unknown. Within my time and memory, Germany and Italy have been made into great and powerful nations, when, previously, they were but separate, independent, and often warring states. The far East has been opened to western civilisation, and is making rapid strides in its efforts to overtake the West.

Surely, when you come to think of it, the world has moved in a truly marvelous way within very recent times. What wonder, then, that the great and all-important science and art of medicine has felt the onward push, and has gone on with the rest,—has even led the van? Modern medicine may be said to date from the publication of Virchow's great work on Cellular Pathology, in 1858, fifty-two years ago. About that time appeared another book, Darwin's Origin of Species. which in its influence on modern thought, has had no equal. Nor should we forget the number of great men in other walks of life who came into the lime light about that time,—our own Lincoln being the most illustrious.

But there was a great void in our knowledge, until the establishment of the germ theory, by Pasteur and Tyndall, at about the same time, and its application to medicine and surgery by Lister, 1880, and, later, by Koch and others. Probably no discovery of modern times has had and is to have a more far-reaching influence on the human race than this one of Pasteur and his co-laborers. By its later developments, it has revolutionised surgery, made safe operations previously undreamed of, and before impossible. It has greatly reduced the mortality in childbirth. It has, almost to as great a degree, revolutionised the practice of internal medicine, enabling us to conquer certain of the most dreaded diseases which affect humanity,—diphtheria, cerebrospinal meningitis, and hydrophobia; and has placed in our hands methods by which we can stop the ravages of such dread destroyers as malaria, yellow fever, bubonic plague, typhoid fever and other maladies. It has shown us the true nature of tuberculosis, and has pointed out new ways for its successful treatment if taken early, and promises, some day, to bring us a cure.

Not only has the germ theory revolutionised medicine and surgery, but it has affected commerce and agriculture as well. It has shown us how to enrich the soil and to preserve in a healthful condition, certain foods. In fact, a careful study would surprise anyone but an advanced scientist, with the far-reaching effects of the knowledge gained by the discovery of the wonderful power of living germs.

It is in its application to surgical practice that I have been most interested. When I was a medical student, much thought was being given to the origin of pus and the causes of inflammation. All was thick darkness. The theories which followed each other in rapid succession, only to be found wanting and to be thrown aside, now seem to us utterly ridiculous. Even then they were unsatisfactory. A history of the changing pathological theories of fifty down to thirty years ago, would well repay careful study.

When Lister, a name to be reverenced by surgeons everywhere, first applied the germ theory to surgical practice, and showed us that, as germs were the cause of surgical infection, the only way of preventing such infection was by keeping the germs out, he met at first with scant recognition and had few followers. Gradually the brightest young men began to see the light which he had kindled, and began to follow his teaching. Improvements, not in the theory, but in the technic, rapidly followed. Methods, at first cumbersome and difficult to carry out, were simplified and popularised, until the details of modern surgical technic leave little to be bettered.

Let me tell you a little of what surgery was in my days as a medical student and hospital interne. In Bellevue Hospital, New York, at that time an amputation was almost certain death. Hospital gangrene and pyemia, diseases now nearly unknown in a well conducted hospital, were almost sure to attack every case, with a necessarily bad result. Compound fractures, when the end of the broken bone comes through the skin, were nearly as dangerous. In the General Hospital in this city, there was at that time a record of 17 successive cases of compound fracture, all fatal.

Blood-poisoning was so common in hospital obstetric practice that, about 1877, a medical congress in Brussels declared that all lying-in hospitals and wards ought to be abolished throughout the world. During the first year of my residence in Buffalo, it was necessary to close the obstetric ward every few month, and to start again in a new portion of the hospital. But I need not multiply examples. Compare results now. Hospital gangrene is unknown to the present generation of surgeons. Septicemia is rare, and occurs only under conditions where the surgeon is unable to get at its source in the start. Lying-in hospitals are safer today than a well conducted home; while surgical operations of a nature undreamed of by my teachers are done safely every day.

Why these changes, Simply because the surgeon has learned the power of the germs of disease, and has learned how to prevent their ingress into the wounds with which he has to deal. He carefully sterilises everything which may be brought into contact with the wound,—instruments, sponges, ligatures, and, above all, his own hands. It is strange that the hands of a surgeon, without which he could do nothing, should so often have been the bearers of the germs which may cause death. The sterilisation of the hands is a very difficult matter, but the use of rubber gloves seems to accomplish the same result most perfectly. I look upon the use of gloves as one of the greatest advances which has been made in surgery and obstetrics within the last ten years.

In the matter of the introduction of new operations, Americans have had a very important part to play. Abdominal surgery was originated by Ephraim McDowell in the then backwoods of Kentucky, in 1809, 101 years ago. The story of that wonderful event has been told so often, that I must ask your pardon for rehearsing it. Last year we celebrated its centennial. The fact that it was done only one year more than a century ago seems to make the time and opportunity fit for me to give you a short review.

Ephraim McDowell was a surgeon of Danville, Kentucky. He was by no means a rough, untaught backwoodsman, but rather a representative of the best training of his time. It is known that he possessed an excellent library, and devoted much of his time to his books. After studying in this country, he went to Edinburgh, where he worked under illustrious teachers, the renowned surgeon, Mr. John Bell in particular. In some of the lectures he heard it said that it might be possible to successfully remove ovarian tumors,—up to that time an opprobrium to surgery. After establishing himself in practice, and after thinking the matter over for many years, he determined that, if the opportunity ever offered, he would attempt the operation.

At last the chance came. A Mrs. Crawford applied for relief, suffering from this dread malady,—then certain to result in death. McDowell, by this time a skilled, experienced, and locally famous surgeon, explained to her the dangers and the possible result. She resolved to take the chances. It was a brave act, brave on both their parts,—on one side no experience to guide and a certain condemnation if he failed (there is no truth in the story that a mob threatened his life if the result proved fatal); on the other side, no anesthetic to relieve the pangs of the cruel knife; no certainty of a successful result. There was, however, a possibility of a cure, and the salvation from a lingering and painful death. The result was fully successful, as it was in a number of cases operated on later,—twelve in all,—and served to place Ephraim McDowell in the ranks of the immortals.

It was a long time before the operation of ovariotomy was firmly established, for the opposition was tremendous. It was a great innovation and contrary to all the best traditions of the profession. The medical mind is strongly conservative, and at that time prejudice and unreasoning opposition held sway more than they do today.

After McDowell, came as operators, Nathan Smith, the Atlees, Peaslee and Dunlap. The operation continued to be denounced everywhere,—in the medical societies, in the medical colleges, and by the general profession. Even the law was invoked to stop the dangerous practice. The surgeons who dared to do it were called murderers and quacks. "There lives the greatest quack in Philadelphia," said a prominent doctor, pointing to W. L. Atlee's home. But the operation would not die, nor did many of the patients. Gradually in this country, and in England by the help of Clay, Baker Brown and Spencer Wells, it was put on a firm foundation, and with the improved results attained after the adoption of Listerism, all opposition was withdrawn. Thus was abdominal surgery first established by the bravery, skill and intelligence of an American.

Nor is he the only one who has served to add to the fame of American surgery. After the discovery of anesthesia,—another great American feat,—and the germ theory, the improvements in surgery came fairly tumbling over each other. The surgery of fibroid tumors, of the gall-bladder and of the appendix, all American in their origin, (the latter by my friend and classmate, Dr. McBurney), has saved countless lives. So the surgery of the thorax and of the brain, in the development of which our distinguished professor of surgery has played an important part, has added greatly to the advance.

I wish time allowed of my telling what this wonderful fifty years has done for internal medicine. I have already alluded to it, and that must suffice. The advance came later, but is still going on at a rapid rate, and bids fair even to surpass the advance in surgery. The removal of a diseased organ is not its cure, and might be likened to the removal, instead of the filling of, a tooth with a small cavity. Though the patient may be symptomatically cured in each case, in neither is the ideal result obtained. It now seems probable that, before long many of these germ diseases will be so certainly curable by medicine, that the necessity for the surgeon will be largely done away with.

Now, let me ask your attention to another topic,—something which has also come under my observation, with which I have been intimately connected and in which I have been greatly interested. I refer to medical education. When I graduated at the College of Physicians and Surgeons, Columbia, New York,—then considered one of the best schools in the country,—the course lasted two years, the term being only sixteen weeks. I went through the course without ever looking through a college microscope, handling a test tube, or entering a laboratory,—unless the dissecting room might be so called,—or examining a case in a hospital ward. When I came to Buffalo, in 1882, much the same state of affairs existed. The course lasted but two years of twenty weeks each, with no entrance examination. There were eight professors, four lecturers, and one professor for all the specialties. There was no attempt at a graded course, the student listening to the same lectures two years in succession. There were no laboratories, nor was there any place in the old college building where proper laboratories could have been established. Four clinics a week, two medical and two surgical, were held in the General Hospital, but students did not enter the wards nor have opportunities to make physical examinations.

The examinations were purely oral, and a diploma obtained under such conditions was a license to practise. Such a state of things seems almost incredible to the student of today. Compare such a course with the one we give now,—a four-year graded course of twenty-eight weeks each year, only to be entered upon after a high school course; the first two years given up mostly to laboratory work, and the last two years occupied largely with clincal instruction. The faculty of eight has increased to about seventy, while ward work in both medicine and surgery has been introduced and enlarged. In obstetrics, after many years of effort, cases are now secured for all the class, and attendance on such cases is now made a necessary qualification for graduation.

Compare. also, the present building with the old affair on Main Street; and even this is so far outgrown that the faculty have it seriously in mind to erect a laboratory building in the immediate future.

Why all this advance? Is it confined to the University of Buffalo? The advance here is only a part of the general movement all along the line. Just as medicine, surgery and obstetrics have advanced, so has medical education followed suit, and the same is true of those cognate sciences,—dentistry and pharmacy. The amount of knowledge to be imparted to the aspirant for medical knowledge now is so great, that two courses of twenty weeks would not begin to suffice.

This development has not been attained suddenly. The first step was taken in 1879, when the school year, previously sixteen weeks, was lengthened to twenty weeks. This was increased to twenty-two weeks in 1882;twenty-three weeks in 1887; twenty-five weeks in 1888, and twenty-eight weeks in 1892, when the new college building was opened. The increase in the number of the faculty has also been gradual; but the great step in enlarging the curriculum, lengthening the school year and increasing the course to a four-year graded course, all date from the occupation of the new building, in 1892, which made these advances possible.

Fault is continually found with our system of medical education. If there be any fault, it must be laid at the door of the profession and the public. The medical school will go on just as far, and just as fast, as it is encouraged to go by the demands of the profession, and just as much as the means placed in its control will allow.

It seems to me that the outlook in general is very encouraging. Already there is considerable talk of a five-year course. Best of all, many of our medical colleges are receiving large endowments. This shows that the laity is beginning to appreciate the value of well educated, well trained physicians. Lately there has been some very severe criticism, by a self-appointed critic, of medical education. The critic seems to be entirely ignorant of the history of medical education in this country. Those who have watched the progress of the last twenty years and know what went before, must certainly be greatly encouraged. The diminution of the number of colleges, which has taken place in many states, largely by consolidation; the lengthening of the college course, which is universal; the introduction of the graded system; the marked raising of the standard; the great increase of laboratory and clinical teaching; the increase in hospital facilities; the marked improvement in methods of teaching; the introduction of state boards of examination; lastly, the endowment of medical schools, before alluded to,—all point to a higher and prouder position for the profession in the future.

To this should be added the strong movement for the better recognition of health matters by the national government. So we may take courage and look at the future of medical education with bright anticipation.

What of the future of our own medical school? Can we keep up with our rivals in the general advance? All friends of our school must look at its future with a certain sense of apprehension. It looks now as if the unendowed medical schools were going to have a hard time. Thus far, schools have depended on a few eminent men for reputation, but that will no longer do. We must have something more. The demands of the higher education mean larger expenses,—laboratories and laboratory equipment, more salaried and better paid teachers, devoting their entire time to instruction; lengthening of the curriculum; increased clinical facilities in hospitals, maternity institutions and dispensaries, under the control of the teaching faculty. All this means money, much money. As I have said, some of the medical schools of the country are getting large endowments. Harvard has five million dollars; Washington University, of St. Louis, a like amount. Rush College, Chicago, is backed by the Chicago University and a large endowment. The College of Physicians and Surgeons, New York, is a part of Columbia University, the largest and one of the richest institutions in the country; the University of Pennsylvania, Johns Hopkins, and several others have endowments. Can we compete?

There will doubtless always be a demand for smaller and cheaper colleges; but even they must maintain a high standard. The outlook for us is not so bright as I wish it were; but I hope that our energetic and able Chancellor, when he has firmly established the Arts Department, will take the interests of the Medical Department in hand, and do something great for it, and thus build another monument.

As I see it, the Law and Dental departments are in a somewhat similar condition, so that the Chancellor will have an opportunity to erect for himself, not only one or two, but a whole row of monuments. Each one we will hope to be more massive and beautiful than the last. What an opportunity for a great man!

The only danger to which the profession as a whole is exposed, seems to be found within its own borders. The spirit of commercialism, which seems to pervade society, has raised its evil head among us and, unless we fight it and expel it, it must ultimately stop progress and degrade the profession to a mere trade.

I have recently given forth my views on one aspect of the subject. I refer to the division of professional fees. My ideas are not accepted by a certain portion of the profession. The principal argument against the view which I have advanced, is that its adoption would ruin business. There is no other sound argument which has been advanced; all the others are sophistries. In other words, the opposition claims that they cannot afford to be honest, that bribery and tipping, and the taking of money on false pretences, are necessary to success in the practice of medicine, and especially surgery. Perish the thought. I do not believe it. I do not believe that the profession will ever, as a whole, sink so low.

It cannot be that our noble profession will ever be degraded to the level of a mere business or trade, and that all the altruistic elements will be eliminated from it. I am sure that there are enough good and true men in the profession to eventually down this insidious foe; but the danger is great, and I warn you against it. Have nothing to do with it, no matter how great the temptation. Do not sell your birthright for a mess of pottage.

Dentistry and pharmacy have by no means stood still during the last twenty years. They are both so closely allied to medicine, that the same agencies which have helped the advance of the one, have helped the others too. The Departments of Pharmcy and Dentistry, although younger, are none the less flourishing branches of the university. The Department of Law is also steadily advancing. This branch of education has shown a development allied to that seen in medicine. This is well illustrated by the history of the Harvard Law School. I use this as an illustration because it is one of the oldest in the country, having been established in 1817. The course at first was only eighteen months, and it was not until 1839 that it was increased, and that by an optional course of six months. In 1870 the six months was made obligatory, and in 1877 another year was added. In 1897 a still further advance was made, and a college degree made necessary for entrance. Thirty years ago the whole law school idea was laughed at by many men high in the profession, who contended that the only way to study law was in the office of a practising lawyer. In this respect law was behind medicine. The addition of a fourth year, already announced by Harvard, will put the course of education for the two professions on an equality, for the present at least.

All this illustrates again what I have said about education being in a state of development. All we ask is, give us time and money, and education in this country, not only in the learned professions, but all along the line, will lead the world. It is now not far behind.

Let us look a little more into the future. Just as I believe that in our political life, right and not might will win, so I believe that in matters of the intellect, truth is bound to conquer. As in medicine there is danger ahead, rocks and shoals to be avoided, so in law there are many difficulties to be overcome in order to keep the standard high.

The legal profession has always deservedly stood as one of the great forces making for righteousness in the community. There can be no denying the fact that its position is somewhat threatened by modern tendencies. As in the medical profession, so in the legal, commercialism is the danger. It is not due to the shysters and hangers-on in the profession. The danger seems to involve some of the ablest and most brilliant men at the bar. Rich clients, especially corporations, are able to find men of spendid intellect who are willing, for a consideration, often a large one, to spend their time, not in upholding the right (I mean "right" in the abstract), but, rather, in so interpreting the law and trying to force their interpretation upon the courts, that their clients may accomplish results which, to the non-legal mind, seem morally wrong and which tend to destroy confidence in both the profession and the courts. This is a great danger, and is one cause of the social unrest so often noted. To be in the employ of a corporation was formerly considered an honor. Now the words "corporation lawyer" are coming to have a sinister meaning. As proof of the truth of this, witness the attack, weak,

I admit, recently made upon the appointment to our highest court of so eminent, upright and able a man as our ruler, Governor Hughes. The facts are there; it is for the profession to determine where the fault lies, and to reform, if reformation be necessary.

Another thing in which lawyers must bestir themselves, is in removing the system of delays and hair-splitting technicalities used in the criminal courts. I quote from a recent address by President Taft, himself a lawyer of eminence. He says:

"I grieve for my country to say that the administration of criminal law in all the states of the Union (there may be one or two exceptions) is a disgrace to our civilisation."

It surely reduces our criminal procedure to a farce, when, in this country, only one murderer in one hundred is convicted and punished. Criminals have escaped on the most trivial technicalities, such as leaving out of the indictment the word "the" before state; because the indictment charged the burglar with intent to commit a "theft" instead of a "felony;" because the indictment named a specific, though correct, date instead of "on or about,"—and many others, which to the non-legal mind would seem silly and ridiculous grounds for diverting the ends of justice. What wonder that lynchings are so common! The disgrace seems to belong to the courts and to the criminal procedure as much as to the people. Recently the mayor of a town in Illinois publicly apologized for a lynching by telling of sorely aggravated cases of juries parleying with justice. It is a sorry state of affairs, and tends to lessen the respect for all law, lawmakers and lawyers.

It is to such as you, young men, and you, young women, who will so soon become the leaders that we must look for reforms. Unless you start out with the idea that there is something else in life beside mere material gain; unless you realize that the things of the mind and of the spirit rank higher than those of the material world, then you will fail, or perhaps you will fall, and in falling disgrace the profession to which you belong.

Remember that your educations is only just begun. You have only laid the foundation, and there is much to be done in strengthening your grasp, in sharpening your weapons, for the conflict. Be sure that you start straight, with a high moral standard, and then keep up to it.

Do not fear that you will not find enough to do. There is still an immense amount to be accomplished in the world. There are great unimagined fields of knowledge to be explored and conquered. It is not to be supposed that in fifty years modern science has done everything, or political science reached the end. There is work enough for all. Disease still claims, and always will claim, its victims; but the number of these victims may be reduced. Suffering will always exist, but its pangs may be mitigated. Injustices exist and will exist, but it is for those skilled in law to see that the laws are so framed and executed as to make these injustices fewer.

Try so to live that, when you come near the end, you may look back and see that, though you have made mistakes, though you have left undone many things which you ought to have done, still you have not lived in vain, and that the world has been made a little better by your having lived and worked in it.

Above all, keep a high ideal always before you, an ideal not only for the individual, but for the race. Better to die on the firing line of human progress, than to live and loiter in the rear. Let mankind be richer for your living and, if opportunity offer, nobler for your dying. I will conclude by paraphrasing the thoughts of another. The mere man ceases, but the race goes on in enduring time. Our little hopes fail to be realised, but the larger hope endures and, if it does endure, if it outlasts dynasties and nations, epochs and civilisations, it is because, in every age, there have been heroes who have freely laid their lives upon the altar of the ideal,—whether that ideal was expressed by religion, by philosophy, by art, by science, or in the humble language of the heart. The unconquerable devotion to something beyond self, a faith, a dogma, a noble principle, or, above all, a divine being, this is what gives dignity to human life and continuity to the life of the race.

"Not achievement, however, mighty it may seem in the moment of its birth, or in the slow ages that build upon it, or wear it away beneath their tread,—not achievement, but aspiration brings a crown to life."

37 Allen Street.

